# Differentially methylated regions in T cells identify kidney transplant patients at risk for de novo skin cancer

**DOI:** 10.1186/s13148-018-0519-7

**Published:** 2018-06-18

**Authors:** Fleur S. Peters, Annemiek M. A. Peeters, Pooja R. Mandaviya, Joyce B. J. van Meurs, Leo J. Hofland, Jacqueline van de Wetering, Michiel G. H. Betjes, Carla C. Baan, Karin Boer

**Affiliations:** 1 Neprology and Transplantation, Department of Internal Medicine, Rotterdam Transplant Group, Erasmus MC, Erasmus University Medical Center, Rotterdam, The Netherlands; 2 Department of Internal Medicine, Erasmus MC, Erasmus University Medical Center, Rotterdam, The Netherlands; 3 Endocrinology, Department of Internal Medicine, Erasmus MC, Erasmus University Medical Center, Rotterdam, The Netherlands

**Keywords:** DNA methylation, T lymphocytes, Epigenetics, Cutaneous squamous cell carcinoma, Non-melanoma skin cancer, Solid organ transplantation

## Abstract

**Background:**

Cutaneous squamous cell carcinoma (cSCC) occurs 65–200 times more in immunosuppressed organ transplant patients than in the general population. T cells, which are targeted by the given immunosuppressive drugs, are involved in anti-tumor immune surveillance and are functionally regulated by DNA methylation. Prior to kidney transplantation, we aim to discover differentially methylated regions (DMRs) in T cells involved in de novo post-transplant cSCC development.

**Methods:**

We matched 27 kidney transplant patients with a future de novo cSCC after transplantation to 27 kidney transplant patients without cSCC and studied genome-wide DNA methylation of T cells prior to transplantation. From 11 out of the 27 cSCC patients, the DNA methylation of T cells after transplantation was also examined to assess stability of the observed differences in DNA methylation. Raw methylation values obtained with the 450k array were confirmed with pyrosequencing.

**Results:**

We found 16 DMRs between patients with a future cSCC and those who do not develop this complication after transplantation. The majority of the DMRs were located in regulatory genomic regions such as flanking bivalent transcription start sites and bivalent enhancer regions, and most of the DMRs contained CpG islands. Examples of genes annotated to the DMRs are *ZNF577*, coding for a zinc-finger protein, and *FLOT1*, coding for a protein involved in T cell migration. The longitudinal analysis revealed that DNA methylation of 9 DMRs changed significantly after transplantation. DNA methylation of 5 out of 16 DMRs was relatively stable, with a variation in beta-value lower than 0.05 for at least 50% of the CpG sites within that region.

**Conclusions:**

This is the first study demonstrating that DNA methylation of T cells from patients with a future de novo post-transplant cSCC is different from patients without cSCC. These results were obtained before transplantation, a clinically relevant time point for cSCC risk assessment. Several DNA methylation profiles remained relatively stable after transplantation, concluding that these are minimally affected by the transplantation and possibly have a lasting effect on post-transplant cSCC development.

**Electronic supplementary material:**

The online version of this article (10.1186/s13148-018-0519-7) contains supplementary material, which is available to authorized users.

## Background

The risk of developing cancer is markedly higher in organ transplant patients than in the general population [1]. The most common cancer in transplant patients is non-melanoma skin cancer whereby cutaneous squamous cell carcinoma (cSCC) occurs most frequently [2], with an increased risk of 65–200 fold [2–4]. Not only the incidence of cSCC increases after organ transplantation, the skin cancer also behaves more aggressively. Transplant patients experience more metastasis and more recurrence of the cSCC: 70% of the patients develop a subsequent skin cancer within 5 years [5, 6]. Identification of transplant patients at increased risk for cSCC may allow early intervention and will improve the quality of life for these patients.

Transplant patients are at high risk for cSCC because of their impaired immune system due to lifelong immunosuppressive therapy [7–9]. Immunosuppressive drugs used after organ transplantation suppress T cell activity [10]. T cells are an important cell type for anti-tumor immune surveillance (CD8+), but can also provide a more immune-tolerant environment for the tumor (regulatory T cells) [11, 12]. Carroll et al. [13] showed that high numbers of peripheral regulatory CD4+FOXP3+ cells predicted the development of a new cSCC in kidney transplant patients who had a previous cSCC. Also the presence of CD8+CD57^hi^ cells, a phenotype associated with T cell senescence, was shown to predict development of a subsequent cSCC in kidney transplant patients [14]. These studies both predicted recurrence of the cSCC; tools to predict de novo cSCC after transplantation are currently unavailable.

Considering the recurrent nature of cSCC and the increased incidence in immunocompromised transplant patients, we hypothesized that there is a systemic defect in patients who will develop cSCC due to an altered state of T cell function. Such an altered state of T cell function is a well-known consequence of loss of kidney function [15]. T cell function is determined by the chromatin state of its DNA, which is a combination of epigenetic features such as DNA methylation, DNA accessibility, histone modifications, and RNA expression [16, 17]. DNA methylation is an important epigenetic regulator of cellular function [18, 19], and high methylation in the transcription start site (TSS) of a gene is in most cases associated with transcriptional silencing of the corresponding gene [20].

Differential DNA methylation between transplant patients with or without a future post-transplant cSCC might provide insight in the pathogenesis of cSCC. However, DNA methylation is a dynamic feature and significantly influenced by the environment [21]. After kidney transplantation, immunosuppressive therapy is given and the metabolic complications associated with loss of kidney function largely disappear. Therefore, it can be expected that changes in DNA methylation will occur and this may also affect any DNA methylation profiles identifying patients at risk for de novo post-transplant cSCC. By comparing these DNA methylation profiles before and after transplantation, the extent of their functional effect on post-transplant cSCC development could be assessed.

In this retrospective study, we aimed to identify kidney transplant patients at risk for de novo post-transplant cSCC by studying genome-wide DNA methylation of T cells. We analyzed samples collected before transplantation and compared patients with a future de novo post-transplant cSCC to patients without cSCC. Highly enriched T cell populations were isolated from these patients and genome-wide DNA methylation was measured. We then searched for differentially methylated regions (DMRs) by comparing the future cSCC patients’ methylation profiles to the non-cSCC profiles. For a subset of cSCC patients, a post-transplantation sample was available which enabled us to compare DNA methylation before and after transplantation. A technical validation of the raw methylation values on the array was performed with pyrosequencing.

## Methods

### Patient samples

Anonymized biobank samples were used in this study; this approach had been approved by the local ethical committee (MEC-2015-642). Kidney transplant patients with a future post-transplant cSCC were matched to kidney transplant patients who have not developed an cSCC based on gender, age (± 2 years), ethnicity, cytomegalovirus (CMV) status, and availability of biobank material. We included patients with at least one cSCC after transplantation and patients with cSCC in situ (Bowen’s disease). Patients with a previous kidney transplantation or another donor organ such as liver, heart, or lung were excluded, as well as patients with a history of malignancy prior to transplantation. Non-cSCC patients with actinic keratosis, a pre-cancerous lesion, were excluded.

The patient cohort consisted of 27 cSCC patients and 27 non-cSCC patients who had been transplanted between 1997 and 2014. No statistical differences were found between the clinical characteristics of the cSCC and non-cSCC patients; however, after cell sorting, the composition of CD4+ and CD8+ T cells significantly differed between the cSCC and non-cSCC patients (Table 1). One cSCC patient had received immunosuppressive drugs prior to an AB0-incompatible transplantation.

**Table 1:** Patient characteristics

	cSCC	Non-cSCC	
	*N* = 27	*N* = 27	
Age (years)^a^	61.7 (27–77)	61.3 (27–77)	*p* = 0.802
Gender (male)	19 (70.4%)	19 (70.4%)	*p* = 1
Years between Tx and first cSCC^a^	5.4 (0.9–12.5)	–	–
CMV status			*p* = 0.46
Negative	12 (44.4%)	9 (33.3%)	
Positive	15 (55.6%)	17 (63.0%)	
Unknown	–	1 (3.7%)	
Dialysis pre-transplantation			*p* = 0.783
Yes	16 (59.3%)	15 (55.6%)	
No	11 (40.7%)	12 (44.4%)	
ESRD diagnosis			*p* = 0.058
Polycystic kidney	6 (22.2%)	1 (3.7%)	
Hypertension	6 (22.2%)	3 (11.1%)	
Diabetic nefropathy	1 (3.7%)	6 (22.2%)	
Glomerulonefritis	3 (11.1%)	6 (22.2%)	
Other	11 (40.7%)	11 (40.7%)	
% CD3^a^	97.4 (92.4–99.5)	98.0 (95.1–99.5)	*p* = 0.225
% CD4^a^	73.0 (45.1–91.4)	60.3 (34.8–80.7)	*p* = 0.000
% CD8^a^	20.7 (5.8–46.2)	32.8 (14.8–60.6)	*p* = 0.000

^a^Median and range

*cSCC* cutaneous squamous cell carcinoma, *CMV* cytomegalovirus, *ESDR* end-stage renal disease

From 11 cSCC patients, material collected after transplantation was available for a longitudinal analysis; characteristics of this subset of patients are given in Table 2. The post-transplantation samples were collected based on the availability of biobank material and are therefore at different time points after transplantation (Table 3). Three of the post-transplant samples were taken after the diagnosis of the first cSCC. All of these patients received treatment, patient “p1” was treated with a topical chemotherapeutic agent 5-fluorouacil, patient “p2” was treated with photodynamic therapy and surgical excision, and patient “p4” was treated with a surgical excision.

**Table 2:** Patient characteristics longitudinal analysis

	*N* = 11
Age at Tx (years)^a^	65.4 (47–75)
Gender (male)	8 (72.7%)
Years between Tx and first cSCC^a^	2.6 (1.1–11.5)
Years between Tx and post-Tx sample^a^	2.1 (0.3–13.0)
CMV acceptor	
Negative	4 (36.4%)
Positive	7 (63.6%)
CMV donor	
Negative	7 (63.6%)
Positive	4 (36.4%)
HLA mismatches^a^	2 (0–6)
Type of immunosuppression directly after transplantation	
Corticosteroids	10 (90.9%)
Tacrolimus	10 (90.9%)
MMF	10 (90.9%)
Cyclosporine	1 (9.1%)
Sirolimus	1 (9.1%)
Basiliximab induction	3 (27.3%)
ATG induction	1 (9.1%)
ESRD diagnosis	
Polycystic kidney	5 (45.5%)
Hypertension	1 (9.1%)
Other	5 (45.5%)
Dialysis pre-transplantation	
Yes	8 (72.7%)
No	3 (27.3%)

^a^Median and range

*cSCC* cutaneous squamous cell carcinoma, *CMV* cytomegalovirus, *ESDR* end-stage renal disease

**Table 3:** Time points longitudinal analysis

Patient	Time after Tx (y)	Time between Tx and first cSCC (y)	Comment
p1	13.0	11.0	Material obtained after diagnosis of first cSCC
p2	7.7	4.1	Material obtained after diagnosis of first cSCC
p3	6.9	7.7	
p4	3.4	2.4	Material obtained after diagnosis of first cSCC
p5	0.9	4.7	
p6	2.1	2.6	
p7	0.3	1.6	
p8	1.1	2.0	
p9	1.1	1.1	
p10	0.6	2.2	
p11	5.0	11.5	

*Tx* transplantation, *cSCC* cutaneous squamous cell carcinoma, *y* years

Peripheral blood mononuclear cells (PBMCs) were isolated by density gradient centrifugation using standard Ficoll-Paque procedures (GE Healthcare, Chicago, IL, US). Isolated PBMCs were stored at − 140 °C until further use. T cells were isolated from the PBMCs using fluorescence-activated cell sorting (FACS) by the BD FACSAria™ ll (BD Biosciences, San Jose, CA, US). PBMCs were stained with anti-CD3 Brilliant Violet 510 (Biolegend, San Diego, CA, US), anti-CD4 Pacific Blue (BD Biosciences), and anti-CD8 APC-cy7 (BD Biosciences), and to exclude nonviable cells, 7AAD PerCP (BD Biosciences) was used. After cell sorting, the purities were > 92% for CD3+ cells; samples below 90% were excluded for further analysis.

Before isolating DNA from the T cells, all patient samples were randomized to minimize batch effects. DNA was isolated using the QIAamp DNA Micro kit (Qiagen, Venlo, The Netherlands) according to the manufacturer’s protocol. Purity and concentration of the isolated DNA was assessed with the NanoDrop ND-8000 (Isogen Life Science, Utrecht, The Netherlands). DNA degradation was determined by gel electrophoresis; none of the samples showed significant degradation.

### DNA methylation microarrays

To generate genome-wide DNA methylation data, 500 ng of genomic DNA was treated with sodium-bisulfite to induce methylation-dependent changes in the DNA sequence, using the EZ DNA Methylation kit (Zymo Research, Irvine, CA, US). DNA was then hybridized on Infinium HumanMethylation450 arrays (Illumina, San Diego, CA, USA) according to the manufacturer’s protocol, and IDAT files were generated by the iScan BeadChip scanner (Illumina).

Data quality was examined using the MethylAid R package [22, 23]. All samples passed the five quality controls performed using the default MethylAid thresholds. Probes with a detection *p* value > 0.01 were removed from the dataset as well as probes containing single nucleotide polymorphisms. Since our patient population was a mixture of male and female, all probes on the sex chromosomes were also removed. A between-array normalization was applied to the type Ι and type ΙΙ probes separately using the DASEN method within the wateRmelon Bioconductor R package [23–25]. The methylation level for each cytosine-phosphate-guanine (CpG) site was calculated as the ratio of the methylated probe intensity and the overall intensity. This is presented as a beta-value, a value between 0 (unmethylated) and 1 (fully methylated). After the quality controls and normalization, beta-values of 423,289 CpG sites remained for further analysis. Both the raw and normalized data are available via the NCBI Gene Expression Omnibus (GEO) database with accession number GSE103911.

### Data analysis DNA methylation microarrays

To identify DNA methylation differences between the future cSCC and non-cSCC patients, we fitted a linear mixed effect model using the lme4 R package [26]. The fixed effects included age, percentage CD4, percentage CD8, and CMV status. %CD4 and %CD8 were included in the model because we found that the composition was different between the cSCC and non-cSCC patients after cell sorting (Table 1). Array IDs were included as a random effect to account for technical variation between the arrays. Single site-specific *p* values were obtained and these *p* values together with the genomic location of the CpG sites were used as input into comb-p [27] to find differentially methylated regions (DMRs).

Comb-p is a command-line tool based on a python library to spatially correlate *p* values [27]. Since DNA methylation at adjacent CpG sites is correlated, it strengthens the data to study regions that are differentially methylated instead of single sites [28, 29]. Comb-p calculates a weighted correlation between the *p* values from the single CpG site-specific analysis and combines adjacent *p* values based on this correlation. A sliding window of 500 base pair (bp) was used, and the seed was set at *p* < 0.01. It then performs a false discovery rate (FDR) adjustment to this new correlation adjusted *p* values, finds regions of enrichment at an FDR cut off of 0.05, and assigns significance to those regions. Multiple testing correction in this analysis is done using a Šidák correction (Šidák < 0.05) [30]. The resulting DMRs were annotated to ROADMAP reference data of primary CD3+ cells [16] to determine the CpG island content and the chromatin state of the DMRs.

### Longitudinal analysis

For 11 cSCC patients (Tables 2 and 3), we compared DNA methylation values of the DMRs before and after transplantation. A paired statistical analysis was done per region. To improve clarity, only those CpG sites within a DMR with a Δbeta-value larger than 0.05 (5% methylation) were used for detailed graphical representation and the patients were evenly divided in four time segments after transplantation. The CpG sites within a region that increased or decreased less than 0.05 in beta-value per patient were considered stable in time.

### Technical validation

Performing methylation arrays for a risk assessment is not easily applicable to clinical practice due to high costs and labor-intensive workflow. Therefore, we tested whether we could obtain the same methylation values with bisulfite pyrosequencing, an easy technique to quantitatively measure single-site DNA methylation [31]. CpG sites within the DMRs 2 and 3 were analyzed in the same DNA samples that were used for the array analysis. Of 10 patients, a mixture of cSCC and non-cSCC patients, 200 ng genomic DNA was bisulfite converted using the EZ DNA Methylation-Direct kit (Zymo Research) according to the manufacturer’s protocol. The bisulfite-treated DNA was then amplified by polymerase chain reaction (PCR) using the Pyromark PCR kit (Qiagen). Primers for PCR and pyrosequencing were designed using PyroMark Assay Design 2.0 software (Qiagen). The PCR primers, melting temperatures, and amplicon sizes for the different PCR products can be found in Additional file 1 together with the specific PCR programs for each DMR.

After confirming the amplicon size by gel electrophoresis, the PCR products were sequenced using a PyroMark Q24 pyrosequencer (Qiagen). Minor adjustments were made to the manufacturer’s protocol: to immobilize the PCR product 1 μL Streptadivin Sepharose High Performance Beads (GE Healthcare) was used per sequence reaction and annealing of the sequence primers was done for 3 min at 80 °C. The sequence primers were added at a concentration of 10 μM. Human high and low methylated DNA (EpigenDx, Hopkinton, MA, USA) were used as controls. DNA methylation percentages were calculated by PyroMark Q24 software (Qiagen).

### Statistical analysis

Differences in characteristics between the future cSCC and non-cSCC patients were statistically tested using SPSS version 21.0 (IBM Corp., Armonk, NY, US). The Mann-Whitney *U* test was used for the continuous variables and *χ*^2^ test for the categorical variables. Data processing and statistical analysis of all the microarray data was done in RStudio version 1.0.136 (Rstudio Inc., Boston, MA, US) with R version 3.2.5 [24]. Cohen’s *D* was calculated on the residuals of the linear mixed effect model by the formula *D* = (mean_cSCC_ − mean_non-cSCC_)/sd_pooled_ in R. Analysis of the differences between methylation in pre-transplantation and post-transplantation samples was done using a paired Wilcoxon ranked sum test using R. Correlation between the DNA methylation levels quantified by pyrosequencing and the beta-values of the Illumina 450k arrays was calculated using Spearman’s rank correlation coefficient using SPSS. All statistical tests were two-tailed, and a *p* < 0.05 was considered statistically significant.

## Results

### Differentially methylated regions

To identify DMRs in T cells between patients who will develop cSCC after kidney transplantation and those without cSSC, we analyzed genome-wide DNA methylation of kidney transplant patients before transplantation. After cell sorting the T cells, we observed a difference in CD4/CD8 composition between the future cSCC and non-cSCC patients’ T cells. The future cSCC patients had a higher percentage of CD4+ cells than the non-cSCC patients (*p* < 0.001; Table 1). For this reason, we included the percentage CD4+ and CD8+ in the linear mixed model as covariates, thereby avoiding potentially biased results with respect to the differences in DNA methylation. None of the single-site *p* values passed the multiple testing correction (Additional file 2: Figure S1); therefore, we continued to DMR analysis.

We found 16 regions significantly differentially methylated between the future cSCC and non-cSCC patients. In Table 4, the genes annotated to the DMRs, the genomic location of the DMRs according to the hg19 genome build (UCSC Genome Browser), and the number of array probes within the regions are presented, and the gene functions are shortly described. Also the Cohen’s *D* is presented per region which is a measure for effect size taking into account the standard deviation in the two groups. Out of the 16 DMRs, 5 were hyper methylated and 11 were hypo methylated in the future cSCC patients.

**Table 4:** Resulting differentially methylated regions of the pre-transplantation analysis

	Genomic location (hg19)	Length DMR (bp)	No. of probes	Regional *p* value	Cohen’s *D*	DMR state
1	chr19:4531638-4531962	324	4	3.57·10^−11^	0.95	Hyper
2	chr5:63461216-63461931	715	10	5.51·10^−10^	− 0.54	Hypo
3	chr3:44753865-44754399	534	11	8.18·10^−10^	− 0.60	Hypo
4	chr2:3699195-3699564	369	5	9.35·10^−10^	0.81	Hyper
5	chr6:168197177-168197700	523	6	6.54·10^−9^	− 0.68	Hypo
6	chr4:165898666-165898968	302	8	1.49·10^−8^	0.54	Hyper
7	chr5:140305947-140306459	512	10	2.38·10^−8^	− 0.53	Hypo
8	chr2:177014555-177015126	571	12	4.35·10^−8^	0.41	Hyper
9	chr1:185703201-185703689	488	12	1.89·10^−7^	− 0.42	Hypo
10	chr6:30698584-30698988	404	11	2.90·10^−7^	− 0.48	Hypo
11	chr19:52391078-52391606	528	12	6.59·10^−7^	0.58	Hyper
12	chr8:54164051-54164443	392	8	1.20·10^−6^	− 0.48	Hypo
13	chr7:51539131-51539584	453	5	1.61·10^−6^	− 0.64	Hypo
14	chr6:88757302-88757704	402	6	1.80·10^−6^	− 0.55	Hypo
15	chr2:74875227-74875549	322	8	1.45·10^−6^	− 0.47	Hypo
16	chr8:96085385-96085690	305	3	1.22·10^−5^	− 0.74	Hypo

*DMR* differentially methylated region, *chr* chromosome, *bp* base pair

### Genomic characteristics of the DMRs

**Fig. 1:**
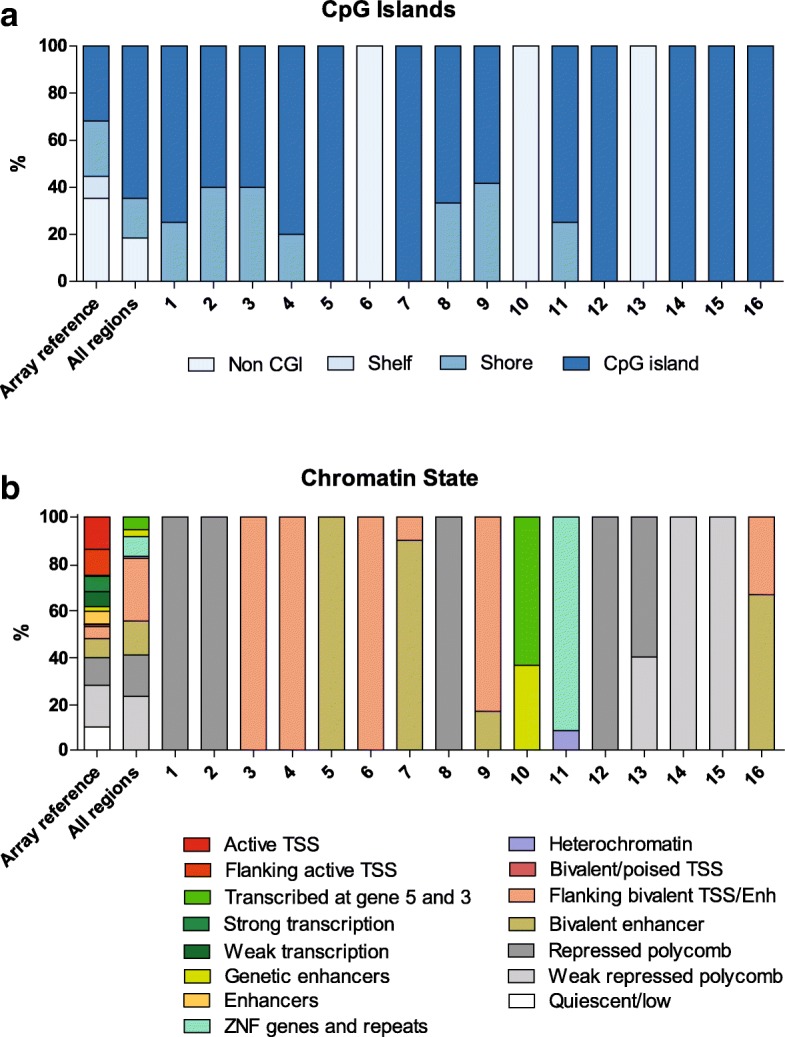
The genomic characteristics of the CpG sites within each DMR. **a** CpG island content for all regions together and the individual DMRs separately, the array content is given as reference. The color represents the CpG island content of each CpG site within that region according to the legend below the graph. **b** Primary T cell-specific chromatin state according to the 15-state model of the ROADMAP epigenomics reference data [16] for all regions together and the individual DMRs separately, the array content is given as reference. The color represents the primary T cell-specific chromatin state of the CpG sites within that region according to the legend below the graph

Since CpG islands are often found near transcription start sites (TSS) and are involved in transcription initiation [32], methylation of CpG islands could have a downstream effect on gene activity. Together with the cell-type-specific chromatin state of the DNA, this could indicate the biological function of a genomic region. In Fig. 1a, the CpG island content is depicted for all regions together and the individual DMRs separately; the array content is given as reference. The 16 DMRs are enriched for CpG islands, slightly less CpG sites are within the shores (< 2 kb flanking CpG islands), and CpG sites within shelves (< 2 kb flanking shores) are absent in these DMRs. For the chromatin state, we annotated the CpG probes within each DMR to ROADMAP epigenomics reference data of primary T cells using the 15-state model [16] (Fig. 1b). Although this might not be an accurate representation of the chromatin state within the T cells we analyzed, it does provide a general perspective on functional and primary T cell-specific characteristics of the genomic region where the DMRs are located. The chromatin states “flanking bivalent TSS/enh” and “bivalent enhancer” are enriched in our results; also 7 out of the 16 DMRs are within repressed or weakly repressed polycomb which is a slight enrichment compared to the array content.

### DNA methylation of the DMRs after transplantation

**Fig. 2:**
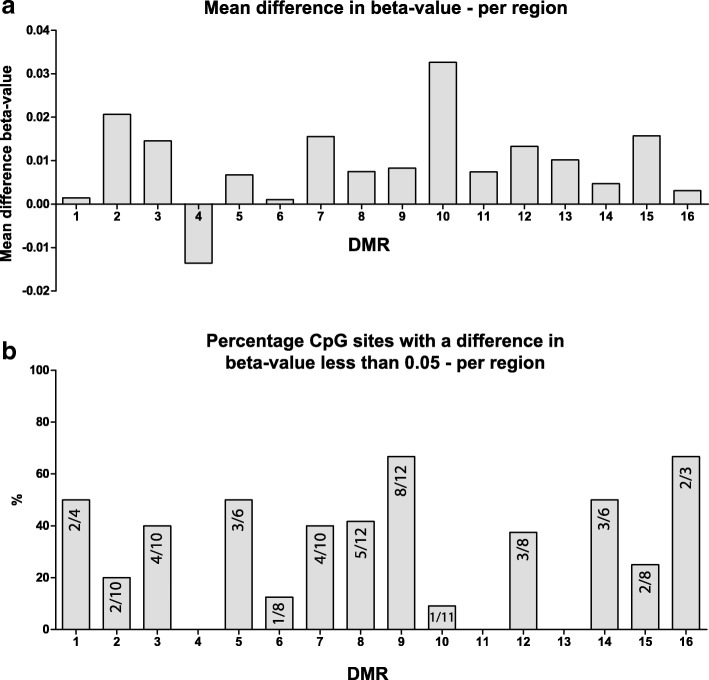
Stability of the 16 DMRs. **a** Mean difference in beta-value per region between pre-transplant and post-transplant samples. The difference is calculated per CpG site for each individual patient and is then averaged over all CpG sites per region for all 11 cSCC patients together. **b** Percentage of CpG sites that show a Δbeta-value of less than 0.05 presented per region. The numbers within each bar represent the number of stable CpG sites from the total sites within that region

To study whether DNA methylation of the 16 DMRs changed after transplantation, we compared beta-values of 11 cSCC patients before and after transplantation. Figure 2a shows the mean difference in beta-value which is an average of all CpG sites per region for all 11 patients together. Overall mean beta-value increased after transplantation. In most regions, there were CpG sites that increased and CpG sites that decreased, therefore showing a mean difference close to zero. All differences in beta-value per DMR and per patient can be found in Additional file 2: Figure S2. A paired Wilcoxon ranked sum test per region resulted in 9 regions that were significantly different after transplantation, after a Bonferroni multiple testing correction (Table 5).

**Table 5:** Results of statistical tests between pre-transplant and post-transplant beta-values per region

DMR	*p* value	Bonferroni correction
1	0.87	
2	1.83·10^−6^	2.92·10^−5^
3	2.03·10^−5^	3.25·10^−4^
4	0.002	0.038
5	0.082	
6	0.55	
7	8.09·10^−8^	1.29·10^−6^
8	0.002	0.033
9	1.51·10^−5^	2.41·10^− 4^
10	3.71·10^−13^	5.93·10^−12^
11	0.028	
12	9.42·10^−5^	0.002
13	0.14	
14	0.32	
15	5.48·10^−5^	8.78·10^−4^
16	0.33	

All CpG sites showed variation within all patients; therefore, to reduce noise and improve clarity, we considered a CpG site that increased or decreased less than 0.05 in beta-value stable. None of the DMRs were 100% stable in time (Fig. 2b); however, some regions showed more stability than others. DMRs 1, 5, 9, 14, and 16 showed at least 50% stable CpG sites whereas in DMRs 4, 11, and 13, none of the sites were stable in time. A more detailed graphical representation of the changes in beta-value per region, per patient, and in time can be found in Additional file 2: Figure S3.

**Fig. 3:**
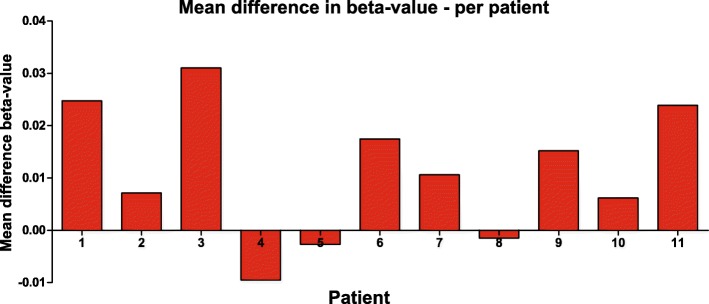
Mean difference in beta-value per patient between pre-transplant and post-transplant sample. The difference was calculated per CpG site for each individual patient and was then averaged over all CpG sites per patient

We also analyzed the mean methylation differences per patient to examine a possible relationship with time after transplantation and with time to clinical onset of the cSCC (Table 3). These mean differences were relatively small in 5 out of 11 patients (Δbeta-value < 0.01) (Fig. 3). Mean methylation differences were not significantly correlated to the time between transplantation and clinical onset of cSCC (*p* = 0.46), nor to time after transplantation (*p* = 0.50), nor to time between post-transplant sample and the clinical onset of cSCC (*p* = 0.09).

### Technical validation

**Fig. 4:**
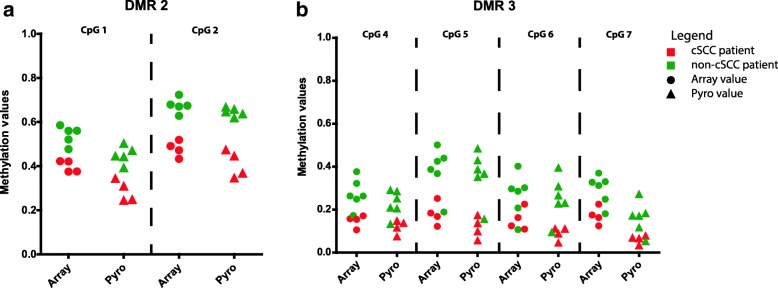
Methylation values on the array and by pyrosequencing of six CpG sites within two DMRs. **a** DMR 2 (*r* = 0.95; *p* < 0.0001). **b** DMR 3 (*r* = 0.88; *p* < 0.0001)*.* The CpG sites correspond to the CpG sites within the DMRs (Table 4)

To confirm the raw beta-values obtained with the 450k array, we performed pyrosequencing analysis of two DMRs (six CpG sites) on the same DNA samples that were analyzed on the array. The DNA methylation values obtained with pyrosequencing were slightly lower than the beta-values obtained with the arrays; this was a consistent deviation across all samples (Fig. 4). There was a strong correlation between the results obtained with the two different techniques; the two sites within DMR 2 had a Spearman correlation coefficient (*r*) of 0.95 (*p* < 0.0001) and the four sites within DMR 3 had an *r* of 0.88 (*p* < 0.0001).

## Discussion

Our results demonstrate that the T cells of patients with a future post-transplant cSCC have different DNA methylation profiles compared to the T cells of kidney transplant patients without cSCC. To our knowledge, this is the first study to show DNA methylation differences in peripheral T cells between patients who develop a post-transplant cSCC and those who do not develop cSCC. In addition, we were able to obtain these results at a clinically relevant time point, before transplantation. The retrospective nature of this study allowed us to carefully match the future cSCC patients to non-cSCC patients and examine the DNA methylation within a highly enriched T cell population.

The observed differences in DNA methylation are predominantly located in CpG islands and bivalent enhancer regions (Fig. 1). Since these are both regulatory genomic regions, it is likely that these differences have a downstream effect in T cells and that differential DNA methylation within these regions could affect T cell function. However, the effect of differential methylation at enhancer regions is difficult to assess since enhancers can regulate genes at large distances in the genome [33]. RNA sequencing would reveal any distal gene regulation by these enhancers; however, that was outside the scope of this study. Here, we focus on the genes that were annotated solely on the basis of close proximity to the DMR.

Out of the 16 DMRs, a few could be associated to cancer by studying literature. Even though these studies were not performed in T cells but mostly in the tumor tissue itself, we can speculate on a possible relationship with post-transplant cSCC development. An example is DMR 11 (annotated to *ZNF577*) which was hypermethylated in our future cSCC patients and showed to be hypermethylated in SCC and adenocarcinoma of the lungs [34]. In addition, an inverse correlation between *ZNF577* gene expression and its DNA methylation was found [35]. DMR 10, which was situated within the actively transcribed gene *FLOT1*, was hypo methylated in our cSCC patients. At first sight, it is an interesting gene due to its involvement in migration of hematopoietic cells [36] and it showed to promote invasion and metastasis of several SCC subtypes when overexpressed [37, 38]. However, in the longitudinal analysis, this was the most varying region (Table 5) with the majority of CpG sites increasing in DNA methylation after transplantation (Additional file 2: Figure S2J). This suggests that this region is greatly influenced by transplantation and it remains unsure how this differential methylation at time of transplantation could affect post-transplant cSCC development.

A kidney transplantation is a procedure with major health effects for an end-stage renal disease (ESRD) patient, and these effects influence DNA methylation. Several studies have shown that blood DNA methylation is associated to kidney function [39, 40]. In addition to that, we showed in a previous study that DNA methylation of T cells can also be modulated by the immunosuppressive medication that kidney transplant patients receive after transplantation [41]. We therefore expected variation between the pre-transplant and post-transplant DNA methylation values in the longitudinal analysis. Indeed, we see that beta-values were significantly different in 9 of the 16 DMRs (Table 4). More interestingly, all but one region increased in mean DNA methylation after transplantation (Fig. 2). This could be a general effect of the transplantation and is in line with findings by Boer et al. [42] showing increased DNA methylation at the *PD1* and *IFNγ* gene 3 months after transplantation.

To determine which regions could have a lasting effect on post-transplant cSCC development, we examined stability of the 16 DMRs after transplantation and considered the CpG sites that stayed within a Δbeta-value of 0.05 stable. DMRs 1, 5, 14, and 16 have 50% or more stable CpG sites and were also not significantly different in a paired statistical analysis (Table 4), suggesting that these differential methylation profiles might have a prolonged effect after transplantation. Considering the possibility of distal gene regulation by these DMRs, their functional effect could be determined by a genome-wide RNA and protein analysis within these T cells. Additionally, to overcome the variability in sampling time points within this study, a prospective study with sampling at regular intervals after transplantation would further assess stability of these DMRs and their function in post-transplant cSCC development.

The development of post-transplant cSCC is the result of a series of events involving different risk factors [2]. Known examples are age, skin type, gender, and possibly immune phenotype [43]. After cell sorting the T cells, we found significantly higher percentages of CD4+ T cells and consequently lower percentages of CD8+ T cells in the future cSCC patients (Table 1). This suggests that an altered CD4/CD8 ratio might be another risk factor for post-transplant cSCC. There is no consensus in literature on the CD4/CD8 ratio in relation to post-transplant cancer development. In contrast to our findings Thibaudin et al. [44] found, over a 10-year observation period, consistently lower counts of CD4+ T cells in patients with future post-transplant malignancy. Although this was not evident at time of transplantation but occurred thereafter. Whereas Bottomley et al. [14] found no significant difference in CD4+ T cell and CD8+ T cell counts or percentages between cSCC and non-cSCC kidney transplant patients.

The relative small sample size in this study is a consequence of selective matching and availability of biobank material. This combined with the single-center design of the study leads to cautious interpretation of the findings. Moreover, we acknowledge that patient pairs can never be perfectly matched. Since we are studying T cells and not skin tissue, where the differences between healthy and malignant tissue are much larger, it was expected that the differences would be subtle. Despite these limitations, the results of this study are a promising first step towards early risk assessment for post-transplant cSCC. To assess the clinical value of these findings, a validation in a different and larger cohort of transplant patients is necessary in addition to our technical validation [45, 46].

## Conclusion

The findings presented here demonstrate the potential of studying DNA methylation of the T cells to identify kidney transplant patients at risk for de novo post-transplant cSCC [47]. We showed that there were systemic differences between future cSCC and non-cSCC patients prior to transplantation. A longitudinal analysis showed that several DNA methylation profiles remained relatively stable after transplantation, suggesting a lasting effect on the development of de novo cSCC after transplantation. In the future, identification of patients at increased risk for post-transplant cSCC before transplantation will allow for early clinical interventions such as regular visits to the dermatologist and stricter lifestyle advice to the patient to minimize additional sun exposure [48]. Ultimately, it may lead to adjustment of the immunosuppressive load but this remains a fine balance between reducing the risk for cancer and causing irreversible damage to the allograft.

## Additional files

Additional file 1: **Table S1.** Sequences of the PCR and sequence primers, amplicon sizes and PCR programs used for the technical validation. (PDF 548 kb)

Additional file 2: **Figure S1.** A Manhattan plot showing all individual CpG sites and their *p* values. **Figure S2A-P.** Differences in beta-value between pre- and post-transplant samples per patient all DMRs. The different dots represent the individual CpG sites within the DMR. **Figure S3A-P**. CpG sites within each region that differ more than 0.05 in beta-value, colored per patient. The y-axis shows beta-value and the x-axis time in years after transplantation. Time points after transplantation are clustered in 0–1 years (*N* = 3), 1–3 years (N = 3), 3–5 years (*N* = 2) and 5+ years (N = 3). (DOCX 2008 kb)

## Abbreviations

bp: Base pair
CMV: Cytomegalovirus
CpG: Cytosine-phosphate-guanine
cSCC: Cutaneous squamous cell carcinoma
DMR: Differentially methylated region
ESRD: End-stage renal disease
PBMC: Peripheral blood mononuclear cells
PCR: Polymerase chain reaction
TSS: Transcription start site
Tx: Transplantation
