# Supplementary material for: Differentially methylated regions in T cells identify kidney transplant patients at risk for de novo skin cancer
Source: Clin Epigenetics. 2018 Jun 18;10:81. doi: 10.1186/s13148-018-0519-7 (PMC6006560; doi:10.1186/s13148-018-0519-7)
Supplement: Supplementary file 1 — Table S1. Sequences of the PCR and sequence primers, amplicon sizes and PCR programs used for the technical validation. (PDF 548 kb) [file 13148_2018_519_MOESM1_ESM.pdf]

Supplementary table

Table S1: PCR primers, sequence primers and PCR programs for technical validation

DMR	Primers (Forward, Reverse and Sequence)	Amplicon size	CpG sites (Illumina ID)
RNF180	F: 5'-GGTGGAATTTTAGGTATAAGAAGGTAA-3'	229 bp	
	R: 5'-biotin-AAACCACAAAAATTATCCCTATAATCTCC-3'		
	S: 5'-ATTTTAGGTATAAGAAGGTAAG-3'		cg17621438 , cg07850154
	**PCR program:** 15 min at 95°C, 45 cycles of 30 s 94°C, 30 s **58°C**, 30 s 72°C followed by 10 min at 72°C		
ZNF502	F: 5'-TTTAGAGGTGGATTGGGGTTAGGATATTA-3'	159 bp	
	R: 5'-biotin-AAATACCTTCTTCTAAAATCCCATAAAA-3'		
	S: 5'-GGATATTAGTTTTAATTTTTGAAAT-3'		cg21672276, cg10263370, cg11003573, cg15687855
	**PCR program:** 15 min at 95°C, 45 cycles of 30 s 94°C, 30 s **58°C**, 30 s 72°C followed by 10 min at 72°C		

F: Forward primer, R: Reverse primer, S: Sequence primer, bp: basepair, min: minutes, s: seconds
