# Supplementary material for: Differentially methylated regions in T cells identify kidney transplant patients at risk for de novo skin cancer
Source: Clin Epigenetics. 2018 Jun 18;10:81. doi: 10.1186/s13148-018-0519-7 (PMC6006560; doi:10.1186/s13148-018-0519-7)

**Additional file 2**

**Figure S1**: A Manhattan plot showing all individual CpG sites and their p-values. On the y-axis the –log_10_ of the p-value is depicted, the genome wide significance line in red is on –log_10_(1.18·10^-7^), and on the x-axis is the genomic location of all the sites, split up in the different chromosomes. The dots in green represent the CpG sites that are in the significant DMRs.


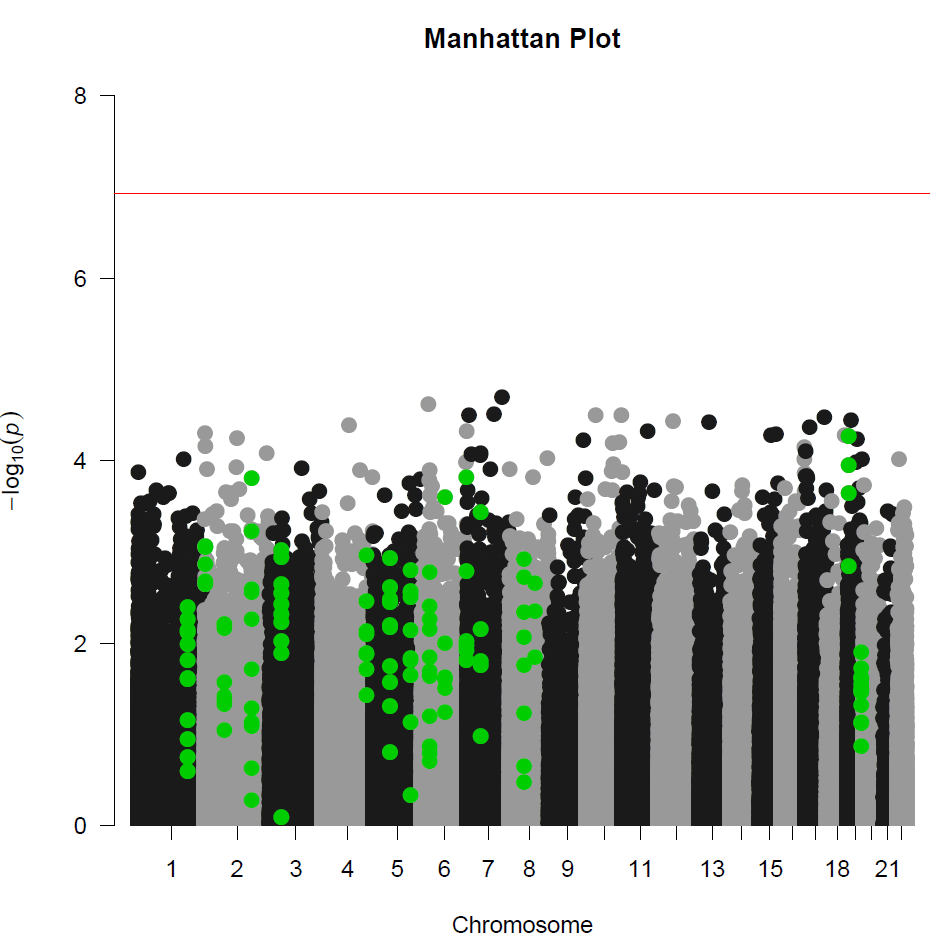


**Figure S2A-H:** Differences in beta-value between pre- and post-transplant samples per patient for DMR 1 to 8. The different dots represent the individual CpG sites within the DMR.

**
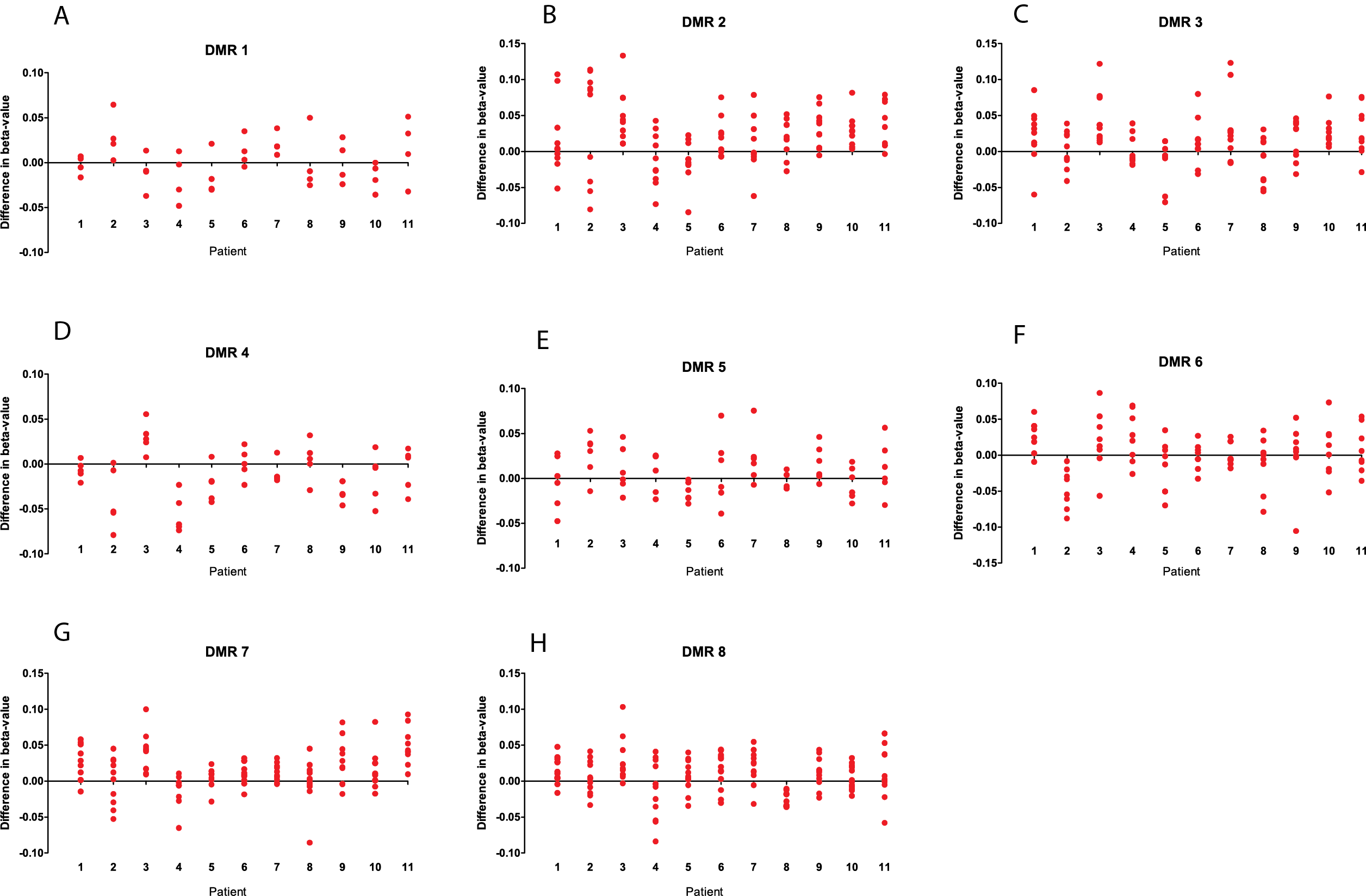
**

**Figure S2I-P:** Differences in beta-value between pre- and post-transplant samples per patient for DMR 9 to 16. The different dots represent the individual CpG sites within the DMR.

**
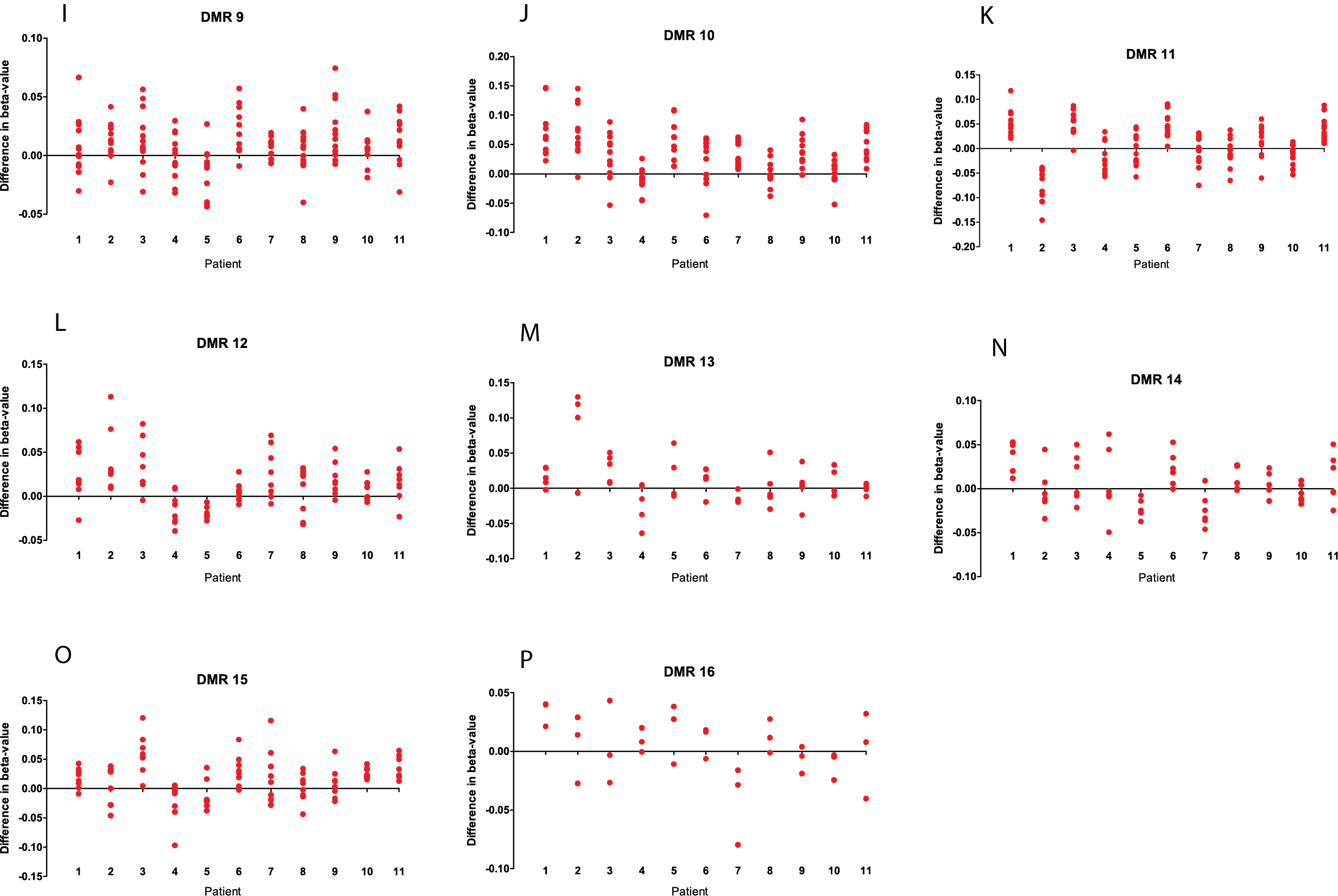
**

**Figure S3A-F**: CpG sites within DMR 1-6 that differ more than 0.05 in beta-value, colored per patient. The y-axis shows beta-value and the x-axis time in years after transplantation. Time points after transplantation are clustered in 0-1 years (N=3), 1-3 years (N=3), 3-5 years (N=2) and 5+ years (N=3).


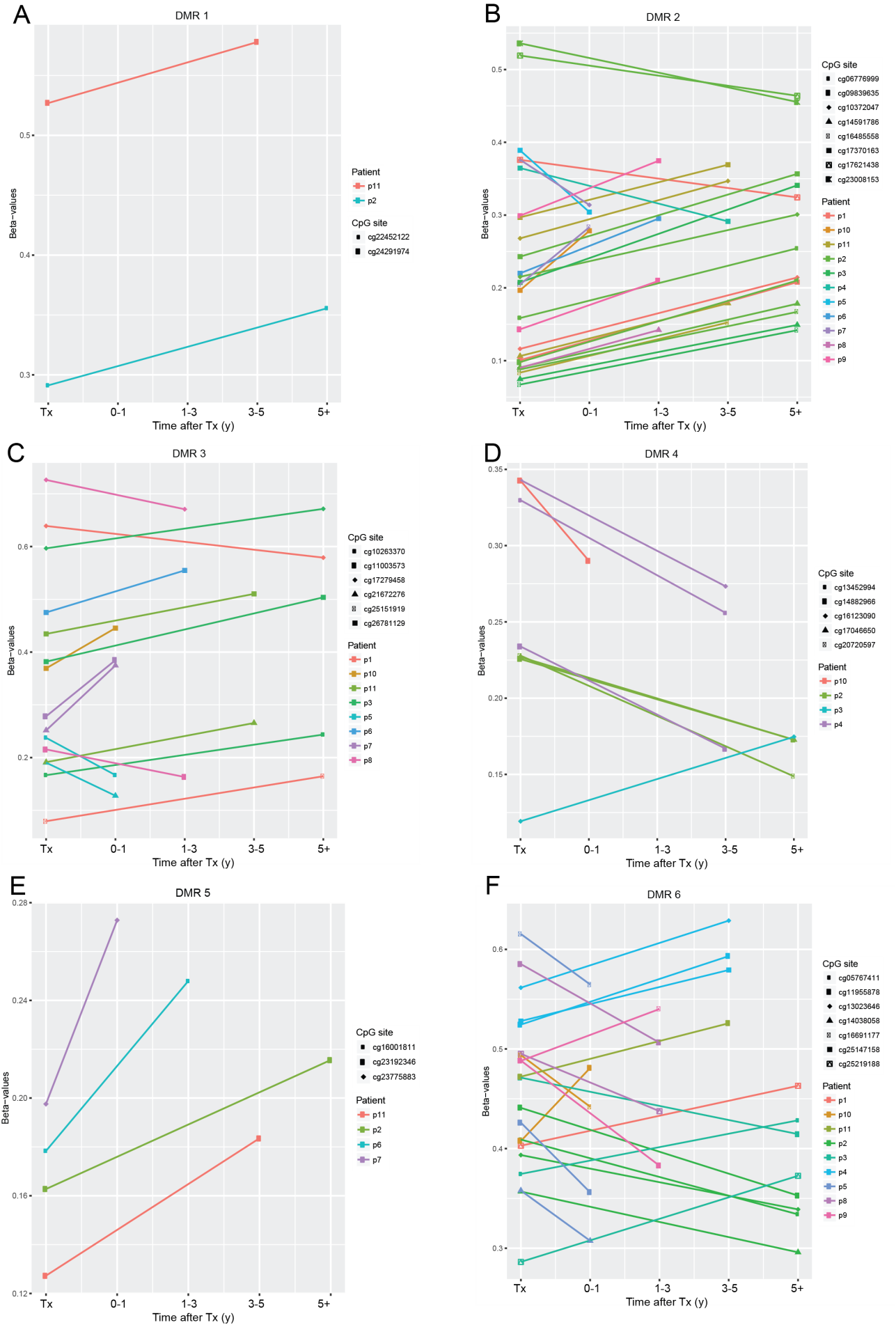


**Figure S3G-L**: CpG sites within DMR 7-12 that differ more than 0.05 in beta-value, colored per patient. The y-axis shows beta-value and the x-axis time in years after transplantation. Time points after transplantation are clustered in 0-1 years (N=3), 1-3 years (N=3), 3-5 years (N=2) and 5+ years (N=3).


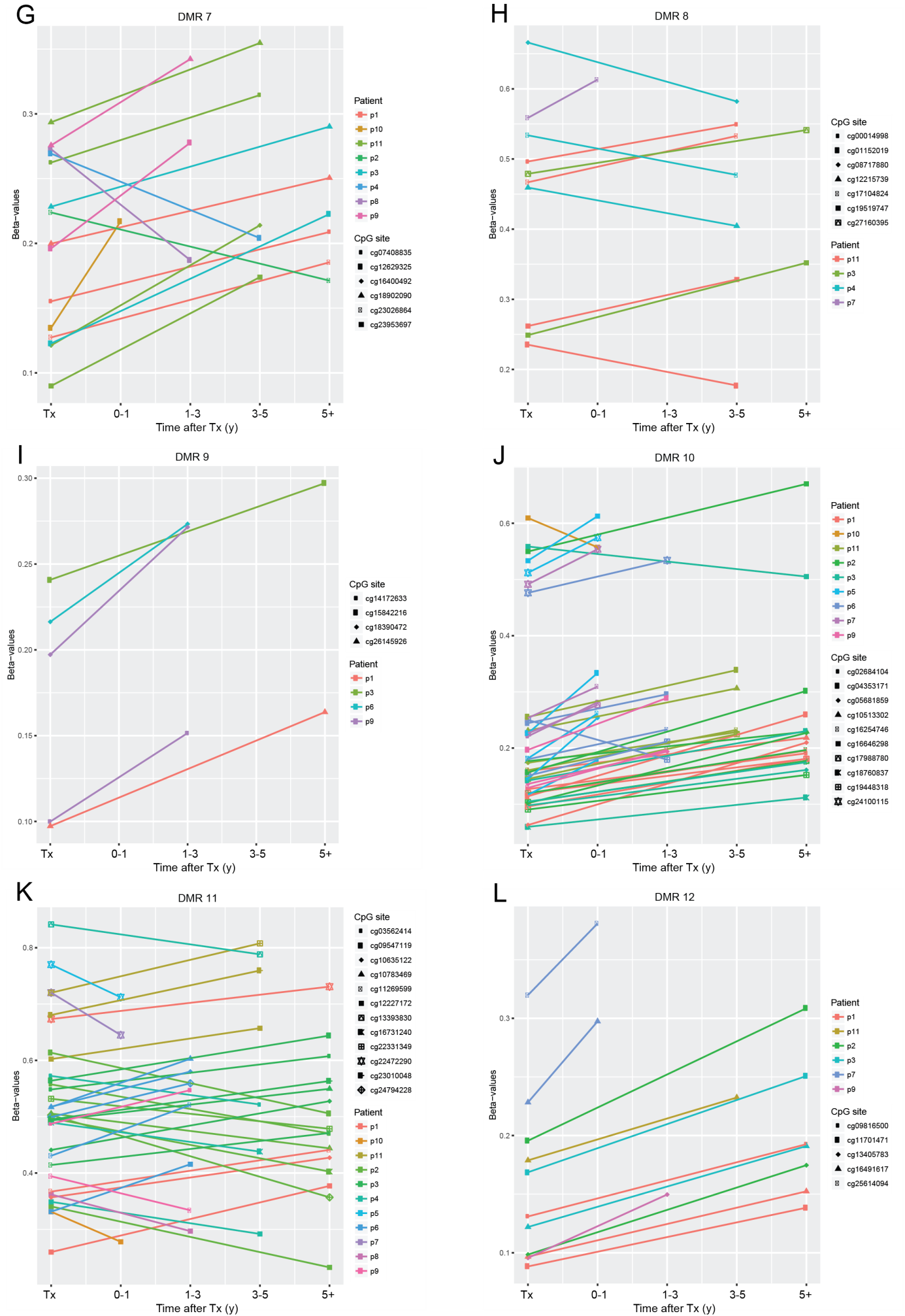


**Figure S3M-P**: CpG sites within DMR 13-16 that differ more than 0.05 in beta-value, colored per patient. The y-axis shows beta-value and the x-axis time in years after transplantation. Time points after transplantation are clustered in 0-1 years (N=3), 1-3 years (N=3), 3-5 years (N=2) and 5+ years (N=3).


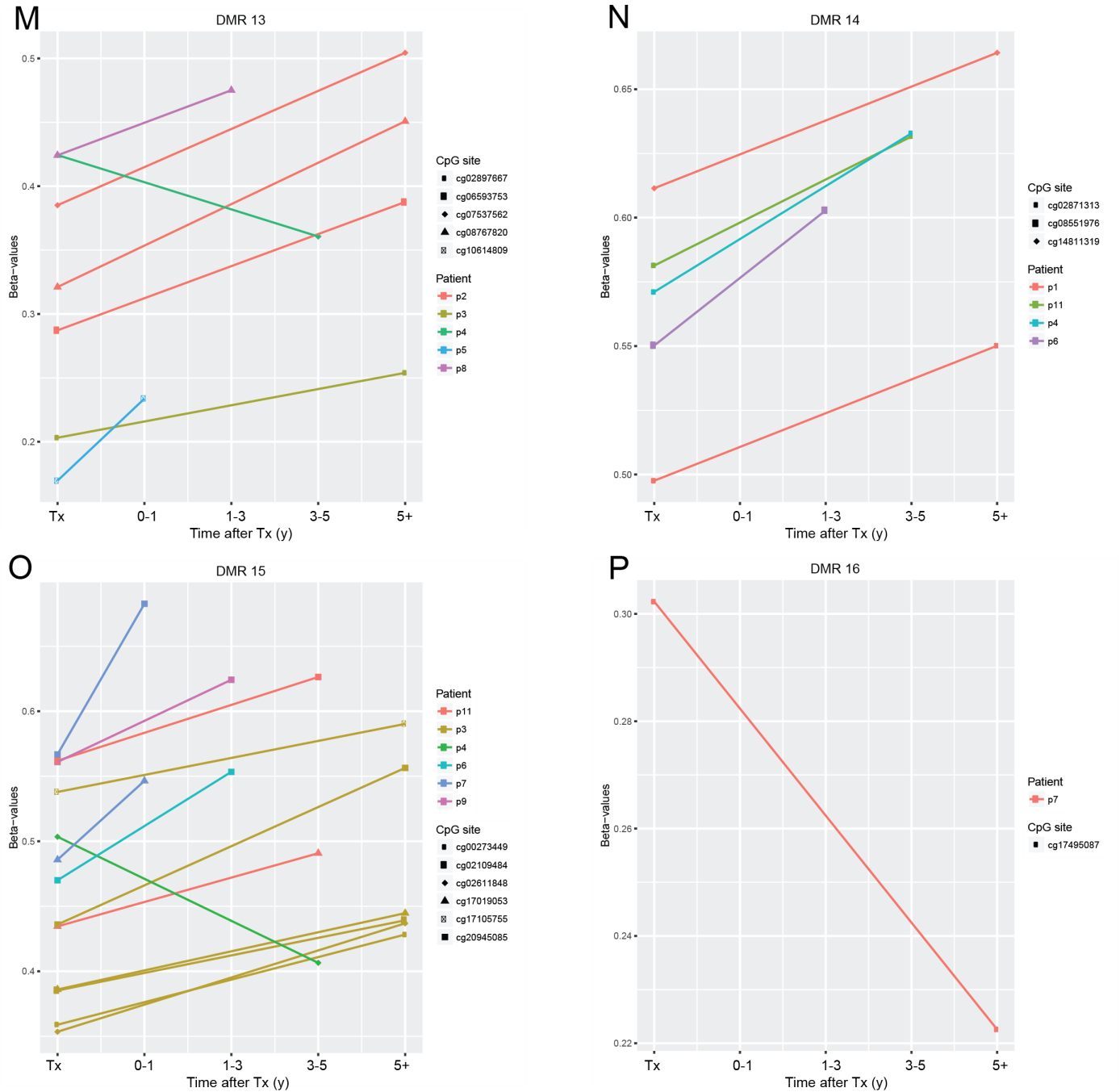

Supplement: Supplementary file 2 — Figure S1. A Manhattan plot showing all individual CpG sites and their p values. Figure S2A-P. Differences in beta-value between pre- and post-transplant samples per patient all DMRs. The different dots represent the individual CpG sites within the DMR. Figure S3A-P. CpG sites within each region that differ more than 0.05 in beta-value, colored per patient. The y-axis shows beta-value and the x-axis time in years after transplantation. Time points after transplantation are clustered in 0–1 years (N = 3), 1–3 years (N = 3), 3–5 years (N = 2) and 5+ years (N = 3). (DOCX 2008 kb) [file 13148_2018_519_MOESM2_ESM.docx]
